# Factors associated with breast cancer awareness and breast self-examination in Fiji and Kashmir India – a cross-sectional study

**DOI:** 10.1186/s12885-020-07583-w

**Published:** 2020-11-10

**Authors:** Rukaiya Malik, Numa Vera, Chandra Dayal, Abhay Choudhari, Jyotishna Mudaliar, Amanda Noovao Hill, Ilisapeci Kubuabola, Ronny Gunnarsson

**Affiliations:** 1 Sydney Children’s Hospital, Randwick, New South Wales Australia; 2 School of Health Sciences, College of Medicine Nursing and Health Sciences, Fiji National University, Suva, Fiji; 3 School of Nursing, College of Medicine Nursing and Health Sciences, Fiji National University, Suva, Fiji; 4 School of Medical Sciences, College of Medicine Nursing and Health Sciences, Fiji National University, Suva, Fiji; 5 Northern Division Scabies Control Project, Labasa Fiji by the Murdoch Children’s Research Institute (MCRI), Melbourne, Australia; 6 Pacific Research Center for Prevention of Obesity and Non-Communicable Diseases, College of Medicine Nursing and Health Sciences, Fiji National University, Suva, Fiji; 7 Primary Health Care, School of Public Health, Institute of Medicine, the Sahlgrenska Academy, University of Gothenburg, Gothenburg, Sweden; 8 Region Västra Götaland, Research and Development Primary Health Care, Research and Development Center Södra Älvsborg, Boras, Sweden

**Keywords:** Breast cancer, Breast cancer awareness, Breast self-examination, Breast awareness, Education

## Abstract

**Background:**

In low-income countries breast cancer awareness (BCA) is essential to reduce the proportion of advanced stage presentations of breast cancer. There is a lack of studies using multivariable techniques to explore factors related to BCA in low-income countries. The objective of this study was to identify to what extent women in Fiji and Kashmir, India have BCA and practice breast self-examination (BSE) as well as factors associated with BCA and BSE.

**Methods:**

A survey of women aged ≥18 years was conducted in Fiji and Kashmir, India to assess BCA and rates of BSE. Comparison between Fiji and Kashmir was done using student’s t-test for continuous data and chi-square for binary data. Factors associated with BCA and BSE were analysed using a multivariable logistic regression for Fiji and Kashmir separately.

**Results:**

Data were collected from 399 and 1982 women in Kashmir and Fiji, respectively. Of 1968 women in Fiji 57% were deemed to have an acceptable BCA compared to only 7.3% of 395 women in Kashmir. Having some education was associated with having BCA with an odds ratio of 4.7 (1.7–13) in Fiji and 10 (1.7–59) in Kashmir. Of 1976 women in Fiji 40% had tertiary education while 40% of 392 women in Kashmir had no education at all. The marital status was similar in both samples (*n* = 1973 and 395) with 68–69% being married and 21–26% being single. The lack of female doctors or nurses with whom to discuss issues, was perceived as a problem in both countries.

**Conclusions:**

The key finding is an association between having any level of education and BCA. This correlation was much stronger than for a family history of breast cancer and BCA. Hence, general education to illiterate women may reduce the proportion of women in low-income countries presenting with advanced-stage breast cancer.

**Supplementary Information:**

The online version contains supplementary material available at 10.1186/s12885-020-07583-w.

## Background

Breast cancer has a worldwide incidence of 2.1 million women per year [1]. According to the World Health Organisation in 2018, approximately 15% of all cancer-related deaths among women were due to breast cancer [1]. Breast cancer screening in high-income countries is conducted through mammography. Research has shown that breast self-examination (BSE) is not an effective screening method and does not reduce mortality caused by breast cancer [2]. Hence within high-income countries where mammography or even regular clinical breast examinations (CBE) performed by staff with adequate education are feasible, BSE is highly discouraged [2].

Breast cancer is also a common and serious disease within low-income countries where screening measures for breast cancer rarely exist due to financial constraints. Few mammography machines are found in most low-income countries, and these are often used to confirm clinically suspected cases of breast cancer. The available financial resources are often used for water sanitation, hygiene and prevention of severe infectious diseases and hence there are limited resources left for screening using mammography or regular CBE performed by staff with adequate education [3]. Subsequently, a high proportion of patients present to hospitals with very advanced stages of breast cancer in many low-income countries [4–6] with subsequent worse prognosis compared to women in high-income countries [6]. In these low-income countries, where screening using mammography or CBE is not available, breast cancer awareness (BCA) and BSE remain cruical.

BCA involves women becoming familiar with the normal shape, size and texture of their own breasts in order to recognize changes and present early to health professionals [7]. International Agency for Research in Cancer (IARC) and World Health Organisation (WHO) have advised that countries should not recommend screening through formal BSE but rather breast cancer programmes should focus on promoting BCA and opportunistic clinical breast examinations at primary health care centres in order to facilitate early diagnosis [2].

The majority of women in rural settings in low-income countries appear to have low BCA [6, 8–10]. Previous studies from low-income countries suggest that BCA and BSE are hindered by some religious beliefs [11], lack of knowledge about BSE [12] and lack of general education [12, 13].

The objective of the present study was to identify to what extent women in low-income countries have BCA and practice BSE. Furthermore, by using multivariable techniques, to identify factors associated with BCA and BSE and their relative importance.

## Methods

This was a pragmatic prospective cross-sectional survey study in Fiji and the Kupwara district in Kashmir, India. These sites were chosen due to both being low income countries with subtropical or tropical climate. Furthermore, personal connections or research collaboration already existed between these sites.

### Inclusion criteria

Women aged ≥18 years consenting to participate and deemed able to understand and respond to the survey.

### Questionnaire

Existing validated questionnaires were deemed to be either too short and rudimentary [14], too long and impractical [15–17] or missing essential questions for the purpose of this study. Hence, a 22 item questionnaire was developed for Kashmir, India (Additional file 1). This questionnaire was adopted for Fiji (Additional file 2). Face validity was ensured by discussing preliminary versions of the questionnaire with researchers in the field. It was also tested on a few women to ensure the questions could be understood. Test-retest reliability was not estimated. Women who could read answered the questionnaire themselves while illiterate women were assisted by a research assistant or college student. Answering a questionnaire typically took 10–20 min.

### Data collection in Fiji

Data collection in Fiji took place between the 1st of July to the 20th of September 2017. In structured settings (villages), specially trained research assistants entered villages accompanied by Zone nurses at a suitable time scheduled in consultation with the village headman. Women were approached by those research assistants, and those who volunteered were recruited, consented, and the questionnaire was administered to them. In unstructured settings (settlements), the team entered settlements accompanied by advisory counsellors. Women were approached and those who volunteered were recruited, consented and the questionnaire was administered. A convenience sample of women within the capital city of Suva was also approached.

### Data collection in Kashmir India

Eighteen BCA-education sessions were held over three weeks, from 24th November to 11th December in 2016, in thirteen different villages within Kupwara District of Kashmir. In five of the villages, the education was held in Anganwadi Centres, which are UNICEF rural women and childcare centres. Within the other eight villages, the education was held in local households or schools opened by principals. Information about these upcoming BCA education sessions was given by word of mouth from well-respected male village elders, Anganwadi staff and school principals.

The questionnaire, written in the Urdu language, was distributed prior to the education session to each participant that verbally consented to participate in the research study. Female college students assisted as Kashmiri verbal translators. These students were fluent in Urdu, Kashmiri and English, and helped in the smaller group break up sessions to assist illiterate participants in completing the questionnaire. The principal investigator at the Kashmiri site (RM) both directly supervised and trained the female college student assistants in appropriate delivery of the research questionnaires prior to the BCA-education session.

Any woman personally recognizing any signs of breast cancer would be offered an immediate CBE performed onsite with a chaperone. Any women with suspicion of having breast cancer would immediately be referred to the nearest health care facility with adequate resources for further investigation and treatment.

### Definitions

For the purpose of this study BCA was dichotomized and acceptable BSA was defined as women that a) perceive they know signs and symptoms of breast cancer and b) believe breast cancer can be detected early and c) believe early detection increases survival and d) would attend a doctor in future if noticing a change in the breast. This information could be retrieved from the distributed questionnaires and resembles the more extensive breast cancer awareness scale [15–17]. For the purpose of this study BSE was dichotomized and acceptable BSE was defined as doing a regular examination of any interval compared to those who don’t.

### Statistical analysis

Descriptive statistics were used for comparing the outcome between Fiji and Kashmir. Students t-test was used for continuous data and chi-square for binary data. The statistical software IBM SPSS version 25 was used for all statistical calculations and the level of significance was set to 0.05.

Factors associated with BCA and BSE were identified for Fiji and Kashmir separately using a multivariable logistic regression.. All multivariable logistic regressions were repeated twice with BCA or BSE as the independent variable.

Independent variables were checked for multicollinearity where a tolerance of < 0.3 or a variance inflation factor (VIF) > 3.3 were considered as unacceptable multicollinearity [18].

All multivariable models were evaluated in a ROC analysis producing an area under the curve (AUC) with its 95% confidence interval and the associated *p*-value. The multivariable logistic regression models were also evaluated using Nagelkerke R-square [19].

## Results

Data were collected from 399 women in Kashmir and 1982 women in Fiji with a mean age of 35 years (Table 1). In Fiji 52.2% of all women came from the three main cities Suva, Lautoka and Nadi while 41.8% came from small cities or rural villages. In Kashmir women came from 13 villages with 10–74 women from each village.

**Table 1:** Characteristics of participants

	==== Fiji (*n* = 1982) ====		==== Kashmir (*n* = 399) ====		*p*-value ^b^
	*n*	Estimate ^a^	*n*	Estimate ^a^	
Age in years:	1955	35 (13) ^c^ 31 (25–41) ^c^	399	35 (14) ^c^ 35 (23–43) ^c^	0.30
Marital status:					3.1 × 10^−10^
Single	415/1973	21%	102/395	26%	(above)
Previously married/divorced	129/1973	6.5%	4/395	1.0%	(above)
Engaged	23/1973	1.2%	15/395	3.8%	(above)
Married	1333/1973	68%	274/395	69%	(above)
Highest level of Education					1 × 10^-∞^
No education	24/1976	1.2%	155/392	40%	(above)
Low Primary school	74/1976	3.7%	15/392	3.8%	(above)
High Primary school	158/1976	8.0%	43/392	11%	(above)
Low Secondary school	333/1976	17%	66/392	17%	(above)
High secondary school	603/1976	31%	65/392	17%	(above)
Tertiary education	784/1976	40%	48/392	12%	(above)
Smoker	359/1982	18%	68/394	17%	0.69
Previously had breast cancer	101/1979	5.1%	2/395	0.51%	0.000042
Family history of breast cancer	270/1976	14%	14/398	3.5%	1.3 × 10^− 8^

^a^ Percent is calculated after first omitting blank responses

^b^ Comparison between Fiji and Kashmir. Students t-test for age. Mann-Whitney U test for Level of education. Chi-square for other variables

^c^ First row is the mean age (Standard Deviation). The second row is the median (interquartile range)

### Descriptive statistics

The marital status was similar in both countries with 68–69% being married and 21–26% being single (Table 1). The level of education was much higher among the Fiji women compared to the Kashmiri women (Table 1). Of 1976 women in Fiji 40% had tertiary education while 40% of 392 women in Kashmir had no education at all (Table 1).

The understanding of breast cancer, including BCA, was much higher among the Fiji women compared to the Kashmiri women (Table 2). Of 1968 women in Fiji 57% were deemed to have an acceptable BCA compared to only 7.3% of 395 women in Kashmir (Table 2). Women in Fiji more often reported having felt a suspicious lump/mass/change in their breast (Table 2), having had breast cancer themselves or having had a family history of breast cancer (Table 1) compared to women in Kashmir.

**Table 2:** Breast cancer awareness (BCA) and relation to health care

	==== Fiji (n = 1982) ====		==== Kashmir (n = 399) ====		*p*-value ^b^
	*n*	Estimate ^a^	*n*	Estimate ^a^	
**Understanding of breast cancer:**					
Ever heard about breast cancer	1771/1979	90%	147/397	37%	3.3 × 10^− 129^
Perceive they know signs and symptoms of breast cancer	1323/1980	67%	69/395	18%	7.1 × 10^−74^
Worried about getting breast cancer	1483/1980	75%	364/393	93%	1.1 × 10^−14^
Believe breast cancer can be detected early	1755/1973	89%	147/397	37%	2.7 × 10^− 124^
Believe early detection increases survival	1717/1975	87%	292/388	75%	3.8 × 10^−9^
Having an acceptable breast cancer awareness ^c^	1115/1968	57%	29/395	7.3%	1.2 × 10^−71^
**Action if suspicion of possible breast cancer**					
Have felt suspicious lump/mass/change in breast before	217/1960	11%	13/399	2.8%	0.000002
Did see a doctor if yes to above question	118/198	60%	10/13	77%	0.22
Would attend doctor in future if noticing change in breast	1803/1968	92%	386/396	98%	0.000048
**Breast self-examination (BSE)**					
Have heard about BSE	1116/1978	56%	177/396	45%	0.000019
Have been taught the technique of BSE	840/1978	43%	58/397	15%	1.5 × 10^−25^
Perform BSE on a regular basis Yes/No	976/1967	50%	144/397	36%	0.000001
Time interval between BSE:					4.0 × 10^−11^
Never	1003/1877	53%	253/373	68%	(above)
Once annually	30/1877	1.6%	1/373	0.3%	(above)
A few times per year	71/1877	3.8%	2/373	0.5%	(above)
Monthly (or close to monthly)	215/1877	12%	7/373	1.9%	(above)
More often than once a month	558/1877	30%	110/373	30%	(above)
**Shyness and wish for a female health care provider**					
Feel shy or reluctant to discuss breast health issues	403/1965	21%	132/398	33%	3.8 × 10^−8^
Usual doctor is female	506/1555	33%	105/396	27%	7.7 × 10^−11^
Would prefer a female doctor to discuss breast health issues	1586/1968	81%	365/393	93%	1.1 × 10^−8^
Feel there is a local lack of female doctors/nurses to discuss this	905/1966	46%	377/391	96%	1.4 × 10^−74^
**Last time visiting a doctor**					8.4 × 10^−22^
Never visited a doctor	330/1972	17%	23/393	5.9%	(above)
More than a year ago	319/1972	16%	22/393	5.6%	(above)
Within a year	493/1972	25%	81/393	21%	(above)
Within a month	830/1972	42%	267/393	68%	(above)

^a^ Percent is calculated after first omitting blank responses

^b^ Comparison between Fiji and Kashmir

^c^ Acceptable breast cancer awareness (BCA) is defined as women that a) perceive they know signs and symptoms of breast cancer AND b) believe breast cancer can be detected early AND c) believe early detection increases survival AND d) would attend doctor in future if noticing a change in the breast

Both women in Kashmir and Fiji would attend a doctor if they noticed a change in their breast (Table 2). However, the local lack of female doctors or nurses with whom to discuss breast changes was perceived as a problem in both countries but more so in Kashmir (Table 2).

As an accidental finding, three Kashmiri women (0.75%), of all 399 participating, expressed they had symptoms of breast cancer. A CBE was performed and they were all referred to the nearest health care facility with adequate resources.

### Multivariable models

The lowest tolerance (0.48) and the highest VIF (2.1) was seen for age in decades in the model only including data from Kashmir. In other models the lowest tolerance was 0.86 and the highest VIF 1.2. Hence, there was no relevant multicollinearity.

There was a strong association between having any level of education and BCA with an adjusted odds ratio of 4.7 (1.7–13) in Fiji and 10 (1.7–59) in Kashmir (Table 3).

**Table 3:** Factors associated with having breast cancer awareness (BCA) ^a^

	======= Fiji ^b^ =======		======= Kashmir ^b^ =======	
	*p*	Adjusted odds ratio (95% CI)	*p*	Adjusted odds ratio (95% CI)
Increasing age (one decade)	0.18	1.1 (0.98–1.1)	0.85	0.95 (0.57–1.6)
Married or engaged	0.93	1.0 (0.82–1.2)	1.0	1.0 (0.37–2.7)
Have some education	**0.0033**	**4.7 (1.7–13)**	**0.010**	**10 (1.7–59)**
Is a smoker	0.27	0.88 (0.69–1.1)	0.77	1.3 (0.25–6.7)
Had breast cancer before	0.18	1.3 (0.88–2.1)	1.0	0.0 (0.0-∞)
Family history of breast cancer	**0.031**	**1.3 (1.0–1.8)**	1.0	0.0 (0.0-∞)
Never visit a doctor	0.31	0.88 (0.69–1.1)	0.076	2.8 (0.90–8.7)
Model validation				
n	1913		373	
Nagelkerke R square	0.015		0.15	
AUC	0.00018	0.55 (0.52–0.58)	0.000042	0.73 (0.65–0.81)

^a^ Breast cancer awareness (BCA) is defined as women that a) perceive they know signs and symptoms of breast cancer AND b) believe breast cancer can be detected early AND c) believe early detection increases survival AND d) would attend doctor in future if noticing a change in the breast. The frequency of this endpoint is presented in Table 2 in the row labelled “Having an acceptable breast cancer awareness”

^b^ Multivariate model for Fiji and Kashmir is multivariate logistic regression

Being married or engaged were strongly associated with performing BSE for Kashmiri women, with an adjusted odds ratio of 2.3 (1.2–4.2), and almost also for Fiji women with an adjusted odds ratio of 1.2 (0.99–1.5) (Table 4).

**Table 4:** Factors associated with performing breast self-examination (BSE) ^a^

	======= Fiji ^b^ =======		======= Kashmir ^b^ =======	
	*p*	Adjusted odds ratio (95% CI)	*p*	Adjusted odds ratio (95% CI)
Increasing age (one decade)	**5.3 × 10**^**−16**^	**1.4 (1.3–1.5)**	0.61	0.95 (0.77–1.2)
Married or engaged	0.065	1.2 (0.99–1.5)	**0.0078**	**2.3 (1.2–4.2)**
Have some education	0.88	1.1 (0.42–2.8)	0.71	1.1 (0.62–2.0)
Is a smoker	0.26	1.2 (0.90–1.5)	0.77	0.91 (0.48–1.7)
Had breast cancer before	0.89	1.0 (0.67–1.6)	0.84	1.3 (0.078–23)
Family history of breast cancer	0.58	1.1 (0.82–1.4)	0.16	2.3 (0.72–7.3)
Never visit a doctor	**8.3 × 10**^**−12**^	**0.40 (0.31–0.52)**	0.41	1.5 (0.60–3.6)
Model validation				
n	1913		374	
Nagelkerke R square	0.091		0.036	
AUC	7.8 × 10^− 29^	0.65 (0.62–0.67)	0.0024	0.60 (0.54–0.66)

^a^ Breast self-examination is defined as the woman states she does a regular examination of any interval compared to those who don’t. The frequency of this endpoint is presented in Table 2 and is the inverse of the row “Breast self-examination (BSE) - Never”

^b^ Multivariate model for Fiji and Kashmir is multivariate logistic regression

Having a family history of breast cancer was only associated with BCA in Fiji with an adjusted odds ratio of 1.3 (1.0–1.8) (Table 3). Furthermore, increasing age and never visiting a doctor was associated with BSE in Fiji with an adjusted odds ratio of 1.4 (1.3–1.5) and 0.40 (0.31–0.52) respectively (Table 4).

### Post hoc effect size calculation

For the endpoint of having acceptable BCA, achieved by a total of 57% in Fiji and 7.3% in Kashmir (Table 2), the sample of 1955 women in Fiji and 399 in Kashmir would allow achieving a statistically significant detection of an odds ratio > 3.6 and 2.6 respectively. Assuming a prevalence of the independent variable any education being 98.8% (Table 2). For the endpoint of having acceptable BSE, achieved by a total of 47% in Fiji and 32% in Kashmir (the inverse of the row “Never” in Table 2) and other assumptions the same would allow achieving a statistically significant detection of an odds ratio > 3.4 and 1.8 respectively assuming the same prevalence of any education (Table 2).

## Discussion

The main finding in this study was a strong association between having some level of education and having BCA. Being married or engaged was associated with performing BSE.

### Breast cancer awareness

A common problem in low income countries with low level of education is fatalism. The likely cause of this is that breast cancer is diagnosed late with fatal outcomes creating the impression that there is no use in seeking medical advice since these women are going to die anyway [3]. Hence, low level of education, fatalism and late presentations becomes a reinforcing vicious circle strengthening the prevailing perceptions and maintaining a very low BCA.

BCA is very important to reduce the high proportion of women presenting with advanced stages of breast cancer [7]. This study demonstrates that women with no education had a very high risk of having a low BCA. Similar findings have been shown previously for low- and middle income contries [20–23]. Interestingly, this study found that having any education has a stronger association to BCA than a previous family history of breast cancer. Perhaps women with education have a greater ability to raise themselves above fatalism and realise that seeking medical advice early may make a difference. This suggests that primary education may play an important role in reducing the perception of fatalism and indirectly promote BCA even when BCA is not the focus of the education. Hence, providing primary school education for women is the first key step to increase breast health in low income countries [24]. This is likely to have many positive benefits including increasing BCA through improved literacy skills.

There is potentially at least some effect of education sessions specifically targeted to increase BCA [25] although it remains unclear if these effects would be seen amongst women in rural settings in low-income countries. Despite this, it is reasonable to develop culturally sensitive and linguistically appropriate programs to promote BCA in low-income countries where screening (mammography or CBE) for early detection of breast cancer is currently not available. These targeted sessions are unlikely to be successful unless they carefully consider the sociocultural context [26].

### Breast self examination

Although there is no solid evidence that instructing women in BSE lowers mortality in low-income countries, it is likely to promote increasing BCA [24]. A link between general educational level and BSE has been described previously [22, 23]. In this study we only found a similar association in Kashmir but not in Fiji. This might be explained by the overall much higher education level in the Fiji sample compared to the Kashmiri sample.

### History of breast cancer

Previous family history of breast cancer has been described as associated with increased BCA [27, 28]. In this study a family history of breast cancer was linked to increased BCA in Fiji but not in Kashmir. It is worth noting that having any education in Fiji was more strongly associated with BCA than a previous family history of breast cancer.

Very few women in Kashmir expressed they had a family history of breast cancer and this might explain why this was not significant in Kashmir. However, breast cancer is one of the leading cancer forms in Kashmir [3]. In this study participants in Kashmir were less educated and had less BCA compared with women in Fiji. Hence, the lack of association between a family history of breast cancer and BCA in Kashmir is likely to reflect low BCA rather than low incidence of breast cancer in Kashmir. It is also reasonable to believe many women in Kashmir were unaware that some of their relatives had breast cancer due to a low awareness of this condition.

### Availability of female doctor or nurse

Having a male staff do a CBE makes women feel unprotected [29]. Women in this study frequently expressed that the lack of female doctors or nurses to discuss sensitive issues was a significant problem. Hence, it is important to consider this issue when planning temporary or permanent programs to promote BCA. However, this might be a difficult challenge [30].

### Limitations

This study used a convenience sample with all its potential limitations. The proportion of women having tertiary education in Fiji was 40% (*n* = 1976) suggesting that the convenience sample from the capital of Suva was quite large compared to samples from rural villages in Fiji. Contrastingly, 40% (*n* = 392) of Kashmiri women had no primary school education, supporting that this sample came from a low-income area. Hence, the samples may not be representative of each country as a whole and that means any comparisons of BCA or BSE between countries should be interpreted causiously.

Further limitations to the running of the project in Kashmir is the political climate and the increased internal conflict in Kashmir during 2016, including the closure of schools for an extended period of time and increased lack of security. As a result, the time for data collection was reduced from eight to three weeks and the intended location of education sessions within schools required to be changed to Anganwadi centres and local village households. The changes in structure of the program and locations for conducting the research questionnaires were discussed with Institutional Ethics Committee of Sher-i-Kashmir Institute of Medical Sciences IEC-SKIMs) and approved. However, this unrest resulted in a smaller sample size in Kashmir.

The Kashmir sample had information about allocation of women to villages. However, five of the villages were represented by less than 20 women. For Fiji more than half of women came from the three main cities. This led us to decide that the quality of data was not good enough to allow using village allocation as a random effect. Adjusting for village as a random effect might have given a different result.

A sample size calculation was not done. Furthermore, non-responders were not registered so an estimate of the response rate could not be done. The purpose of this study was to get a brief estimate and exact point estimates of effect sizes (odds ratios) provided should be interpreted with some caution.

### Importance of this study

This is the first study in Kashmir that incorporates factors of reluctance to discuss breast issues with male physicians or nurses and the lack of female health professionals in rural Kashmir. No similar study has previously been done in Fiji. The multivariable technique allows a relative comparison of the importance of different factors on BCA and BSE and it is noteworthy that having any education seems to be much more important than a family history of breast cancer. This study highlights that lack of any primary school education among women in low-income countries outweighs all other risk factors for low BCA. It even outweighs the risk factor of having a family history of breast cancer.

### Generalisability

While findings highlighted an association between level of education and BCA- they have to be interpreted with caution - however they highlight an important issue for women and BCA in particular women from low income countries who may have limited access to education and perhaps for women with literacy problems regarding BCA.

Kashmir, India and Fiji are countries located in different parts of the world with large cultural differences. Findings in the same direction in both these countries, such as lack of primary school education being strongly associated with low BCA, support each other. Hence, this finding is potentially valid also for other low-income countries. However, the limited sample size, especially in Kashmir, could limit generalisability of findings.

## Conclusions

This study points to the strong correlation between having no education and low BCA. This correlation was much stronger than for a family history of breast cancer and BCA. Hence, a reasonable assumption is that promoting general education to illiterate women and providing specific culturally sensitive education programs targeting BCA has the potential to increase BCA and early presentation of breast changes to a health care professional. Subsequently, general education has the potential to reduce the proportion of women in low-income countries presenting with advanced-stage breast cancer. Furthermore, introduction of targeted BCA education to women combined with CBE when necessary, could lead to increased early detection of breast cancer in low income countries.

## Supplementary Information

**Additional file 1.**

**Additional file 2.**

## Abbreviations

BCA: Breast cancer awareness
BSE: Breast self-examination
CBE: Clinical breast examinations
