# Supplementary material for: Factors associated with breast cancer awareness and breast self-examination in Fiji and Kashmir India – a cross-sectional study
Source: BMC Cancer. 2020 Nov 10;20:1078. doi: 10.1186/s12885-020-07583-w (PMC7654031; doi:10.1186/s12885-020-07583-w)
Supplement: Supplementary file 1 — Additional file 1. [file 12885_2020_7583_MOESM1_ESM.pdf]

Breast Self-examination Questionnaire

The information you provide here will remain completely anonymous. Please provide the most appropriate answer for each of the following questions by drawing a circle around the most appropriate answer.

1. Your age (in years): ....

2. Are you a smoker? Yes No

3. Marital status (Tick one):
   - Single
   - Married
   - Engaged
   - Previously married/ divorced

4. Education (Tick highest level applicable to you):
   - Illiterate
   - Primary (Grade 1-5)
   - Middle (Grade 6-8)
   - Secondary (Grade 9 and 10)
   - Higher Secondary (Grade 11 and 12)
   - Graduate degree/college
   - Post graduate degree

5. a) Have you heard about breast cancer? Yes No
   b) Do you believe that breast cancer can be detected early? Yes No
   c) Do you believe early detection increases chances of survival? Yes No
   d) Are you worried about getting breast cancer? Yes No

6. Are you aware of the signs and symptoms of breast cancer? Yes No

7. Have you previously had breast cancer? Yes No

8. Do you have any family history of breast cancer in your parents, siblings or children? Yes No

9. a) Have you felt a breast lump/mass or noticed changes in your breasts before? Yes No
   b) If yes, did you see your doctor about this? Yes No

10. If you feel a breast lump or notice changes in your breast in the future
    a) Would you go see your doctor? Yes No
    b) Is your usual doctor a female or male? Female Male
    c) Would you prefer to see a female doctor for this issue? Yes No
    d) Do you feel shy and reluctant to discuss breast health issues? Yes No

11. Do you feel there is a lack of female doctors or healthcare nurses you can go to about breast issues locally Yes No

12. Have you heard of breast self-examination? Yes No

13. Have you been taught the technique of breast self-examination? Yes No

14. Do you palpate your breasts on a regular basis? Yes No

a) If Yes, how often? ………………………………………………………………………………………..

15. When was the last time you visited your doctor? (Tick one)

- Within a month
- Within a year
- More than a year ago
- Never

Thank you for completing this questionnaire.
