# Supplementary material for: Factors associated with breast cancer awareness and breast self-examination in Fiji and Kashmir India – a cross-sectional study
Source: BMC Cancer. 2020 Nov 10;20:1078. doi: 10.1186/s12885-020-07583-w (PMC7654031; doi:10.1186/s12885-020-07583-w)
Supplement: Supplementary file 2 — Additional file 2. [file 12885_2020_7583_MOESM2_ESM.pdf]

FNU FIJI NATIONAL UNIVERSITY

JAMES COOK UNIVERSITY AUSTRALIA

ID:
Date of Interview:
Division:
Subdivision:
Area:
Interviewer:

Breast Self-examination Questionnaire

The information you provide here will remain completely anonymous. Please provide the most appropriate answer for each of the following questions.

1. Your age (in years):			
2. Ethnicity			
3. Marital status (Tick one):	• Single/ never married		
	• Married		
	• Engaged		
	• Previously married/divorced/separated		
	• In a relationship but not married/Defector		
4. Education Level (Tick highest level applicable to you):	• Nil		
	• Primary	Up to 6th grade (Class 6)	
		Up to 8th grade (Class 8)	
	• Secondary	Up to 10th grade (Form 4)	
		Up to 12th grade (Form 6)	
		Up to 13th grade (Form 7)	
	• Tertiary		

		Please tick yes or no for the following questions		Yes	No
5.		Are you a smoker?			
6.	a)	Have you heard about breast cancer?			
	b)	Do you believe that breast cancer can be detected early?			
	c)	Do you believe that early detection increases the chance of survival?			
7.		Are you aware of the signs and symptoms of breast cancer?			
8.		Are you worried about getting breast cancer?			
9.		Have you previously had breast cancer?			
10.		Any family history of breast cancer in your parents, siblings or children?			
11.	a)	Have you felt a breast lump/mass or noticed change in your breast before?			
	b)	If yes, did you see your doctor about this?			
12.	a)	If you feel a breast lump or notice changes in your breast in the future, would you go see your doctor?			
	b)	Is your usual doctor whom you visit at nearest health facility a female or male?	Male		
			Female		
	c)	Would you prefer to see a female doctor for this issue?			
	d)	In general, do you feel shy and reluctant to discuss breast health issues?			
13.		Do you feel there is a lack of female doctors or healthcare nurses you can go to about breast issues locally?			
14.		Have you heard of breast self-examination?			
15.		Have you been taught the technique of breast self-examination?			
16.	a)	Do you palpate your breasts on a regular basis?			
	b)	If Yes, how often?			

17.	When was the last time you visited your doctor? (Tick one)	Within a month	
		Within a year	
		More than a year ago	
		Never	
18.	Why do you think women do not palpate their breast or do self-breast examination?		

Thank you for participating in this survey
